# Changes in symptoms of anxiety, depression, and PTSD in an RCT-study of dentist-administered treatment of dental anxiety

**DOI:** 10.1186/s12903-023-03061-4

**Published:** 2023-06-22

**Authors:** Mariann Saanum Hauge, Tiril Willumsen, Bent Stora

**Affiliations:** 1 Faculty of Dentistry, University of Oslo, Oslo, Norway; 2 Oral Health Centre of Expertise, Rogaland, Stavanger, Norway; 3 Oral Health Services Agder, Kristiansand, Norway

**Keywords:** Dental anxiety, CBT, Midazolam, Communication, Anxiety, Depression, PTSD, Sedation

## Abstract

**Background:**

Educating dentists in treatment methods for dental anxiety would increase the patients’ access to treatments that are important to their oral health. However, to avoid adverse effects on comorbid symptoms, involvement by a psychologist has been considered necessary. The objective of the present paper was to evaluate whether a dentist could implement systematized treatments for dental anxiety without an increase in comorbid symptoms of anxiety, depression or PTSD.

**Methods:**

A two-arm parallel randomised controlled trial was set in a general dental practice. Eighty-two patients with self-reported dental anxiety either completed treatment with dentist-administered cognitive behavioural therapy (D-CBT, *n* = 36), or received dental treatment while sedated with midazolam combined with the systemized communication technique “The Four Habits Model” (Four Habits/midazolam, *n* = 41). Dental anxiety and comorbid symptoms were measured pre-treatment (*n* = 96), post-treatment (*n* = 77) and one-year after treatment (*n* = 52).

**Results:**

An Intention-To-Treat analysis indicated reduced dental anxiety scores by the Modified Dental Anxiety Scale (median MDAS: 5.0 (-1,16)). The median scores on the Hospital Index of Anxiety and Depression (HADS-A/D) and the PTSD checklist for DSM-IV (PCL) were reduced as follows: HADS-A: 1 (-11, 11)/HADS-D: 0 (-7, 10)/PCL: 1 (-17,37). No between-group differences were found.

**Conclusions:**

The study findings support that a general dental practitioner may treat dental anxiety with Four Habits/Midazolam or D-CBT without causing adverse effects on symptoms of anxiety, depression or PTSD. Establishing a best practice for treatment of patients with dental anxiety in general dental practice should be a shared ambition for clinicians, researchers, and educators.

**Trial registration:**

The trial was approved by REC (Norwegian regional committee for medical and health research ethics) with ID number 2017/97 in March 2017, and it is registered in clinicaltrials.gov 26/09/2017 with identifier: NCT03293342.

**Supplementary Information:**

The online version contains supplementary material available at 10.1186/s12903-023-03061-4.

## Background

Dental anxiety has a high prevalence, and this combined with a high impact on oral health, constitutes a serious public health challenge [1]. In a 2021 meta-analysis the global estimated prevalence of dental anxiety was 15.3% (95% CI 10.2–21.2) [2], meaning that general practicing dentists are required to handle anxious patients nearly on a daily basis.

Cognitive behavioural therapy (CBT) is recognized as the treatment of choice for specific phobias, including the most severe form of dental anxiety, dental phobia [3]. A concern for adverse reactions in patients if this sort of treatment (involving exposure) is applied by a dentist without support from a psychologist or a psychiatrist has been raised. Both the severity of the condition as well as common psychiatric comorbidities that could complicate treatment have been proposed as arguments against CBT treatment by dentists [4, 5]. In evidently, dentists do recurrently expose their dental anxiety patients to their fears through regular dental treatment. In a study investigating how an invasive dental treatment (wisdom tooth removal) affected patients, pain and frequency of previous traumatic experiences were found to increase the risk for the development of symptoms of anxiety and post-traumatic stress following the procedure [6]. Although dental treatment may carry a risk for psychological adverse effects, it is difficult to find evidence that justify a concern for adverse reactions following dentist-administered dental anxiety treatments. Contrarily, favourable findings have been reported in the few studies that do exist on the subject [7–9]. Vassend and colleagues even found positive effects on general distress after dentist administered treatments of dental anxiety of varying severity [10].

The daily management of patients with dental anxiety in general dental practices often includes the use of sedatives [11]. A hesitancy towards conscious sedation as part of dental anxiety treatment is endorsed by reports revealing no positive long-term effects on dental anxiety levels [12]. Performing conscious sedation in an optimal manner requires good relational skills as argued by Woolley in 2016 [13]. In line with this, studies that systematically combine conscious sedation with basic skills for patient management generate more promising long-term effects, including stable reductions in dental anxiety [14–16]. The evidence-based communication model “The Four Habits Model” [17] is an example of a method that has proved to be a helpful tool also in combination with sedation treatment [7].

Awareness of the importance of adequate communication and functional dentist-patient relationships for treatment outcome in dentistry is growing [18]. Still, reports on how dentists’ relational skills affect the outcome of dental anxiety treatments are few. Clinical communication skills, including empathetic skills, have been identified as important when dental students interact with fearful patients [19]. Yuan et al. proposed that effective patient-dentist interaction may reduce dental anxiety and shame and thus function as a driver for regular dental visiting [20]. Since empathy has been shown to be particularly important to anxious patients [21], anxiety treatments could be particularly sensitive to the dentists’ capability to create bonds with their patients.

Although studies on dentist-administered dental anxiety treatments are promising, little is known about how the presence of comorbid psychological symptoms may influence treatment effect, or how dental anxiety treatment may influence pre-existing problems. Knowledge on how these symptom levels fluctuate during and after dental anxiety treatment by a dentist in general dental practice is also lacking. In addition, we know very little about how the dentist-patient relationship influence outcome of dental anxiety treatments. Accordingly, the present study tested the following hypothesis: 1. High score on comorbid symptoms (anxiety, depression, and PTSD) will not negatively affect the dental anxiety treatment effect measured by the reduction in dental anxiety. 2. No increase in symptoms of general anxiety, depression, or PTSD will be observed following systematic dental anxiety treatment administered by a dentist in a general dental practice. 3. The patient rated dentist-patient relationship will be of importance to the outcome of treatment by D-CBT and Four Habits/midazolam.

## Methods

The trial was approved by REC (Norwegian regional committee for medical and health research ethics) with ID number 2017/97 and it is registered in clinicaltrials.gov with identifier: NCT03293342.

### Data

The data presented in this article was collected in a general dental practice in a rural Norwegian town. A total of 96 patients were admitted to the study between September 2017 and March 2020. Recruitment was conducted by a research assistant. In a baseline session the research assistant provided more detailed information about the trial and also about the public treatment alternative in interdisciplinary teams. Thereafter, the participants signed an informed consent form, completed the questionnaires, and were randomly allocated by the assistant to either the D-CBT (*n* = 48) or Four Habits/Midazolam (dental treatment while sedated with midazolam combined with the systemized communication technique “The Four Habits Model” [17]. Table 1) (*n* = 48) group with a 1:1 ratio. Numbers were randomly allocated to two groups by statistical software administered by the first author, MSH. All available numbers were put in opaque envelopes in a box. Participants chose an envelope and were allocated according to the number inside.

**Table 1:** The four habits model

Habit		Skills
**Invest in the beginning**	**O**	Create rapport quickly
	**O**	Elicit the patients concerns
	**O**	Plan the visit with the patient
**Elicit the patient’s perspective**	**O**	Ask for the patient's ideas
	**O**	Elicit specific requests
	**O**	Explore the impact on the patient's life
**Demonstrate empathy**	**O**	Be open to the patient's emotions
	**O**	Make an empathic statement
	**O**	Convey empathy nonverbally
**Invest in the end**	**O**	Deliver diagnostic information
	**O**	Provide education
	**O**	Involve the patient in making decisions
	**O**	Complete the visit

The table describes the main aspects of the Four Habits Model and the skills required for proper use

The treatment conditions were completed in four (Four Habits/Midazolam treatment condition) or five (D-CBT treatment condition) visits, with a total duration of 300 min in both methods. The methods were detailly described in manuals. An English version of the D-CBT manual along with an e-learning programme in Norwegian is freely available at the Faculty of Dentistry, University of Oslo [22] (as well as in an additional file [Additional file 1]). An e-learning programme for “The Four Habits Model” is also available at the same site, while a written manual for the Four Habits/Midazolam treatment condition is to be found in an additional file [Additional file 2]. The methods had some similarities that are detailed in another Additional file [Additional file 3].

The participating patients were offered two alternatives following the interventions: 1) A referral for dental treatment with a general dental practitioner of their choice at full payment, or 2) A referral for further dental anxiety treatment and free dental treatment at a specialised dental anxiety clinic.

Prior to the study, an emergency plan was made that included access to a local psychiatric centre. This was a precautionary measure in the case that strong and acute psychological adverse reactions would arise in response to the treatment. Data collection and storage was on the TSD (Tjeneste for Sensitive Data) facilities, owned by the University of Oslo, operated, and developed by the TSD service group at the University of Oslo, IT-Department.

#### Sample of patients

The inclusion criteria for the RCT study were as follows: (i) self-reported dental anxiety at a level of severity that affected the participant’s ability to receive dental treatment, and (ii) the ability to communicate fluently in Norwegian. The patients were mainly self-referred (*n* = 75) and recruited through advertisements in local newspapers and in social media (Facebook). Local dentists were also contacted and contributed with referrals (*n* = 25). Admission was at the private dental practice. Four patients did not want to participate. In total, 96 patients participated in the RCT study and were randomly assigned to D-CBT (*n* = 48) or Four Habits/midazolam (*n* = 48). The allocation ratio was 1:1. All of the 82 patients invited to the follow-up study had previously completed treatment under one of the two treatment conditions, D-CBT or Four Habits/midazolam.

In the present study, sample size calculations were done with a significance level of 0.05 (α = 0.05) and a power set to 0.8 (β = 0.20). Sample size was estimated from the DAS values among patients with dental anxiety but without diagnosed odontophobia reported in a Norwegian study conducted by Kvale and colleagues [23]. The mean baseline DAS-score was 15.0 (SD = 3.6) and the relevant between-group differences in outcome was considered 20%. Based on these criteria with expected drop-out of 20%, the sample size was set at 35 in each group. In January 2018 reimbursement of dental treatment for the target group changed. Based on a new estimate of 35% drop-out, the sample size was increased to 51 participants per intervention group. However, due to the onset of Covid-19 it was only possible to include 48 participants in each group.

#### One dentist treated all the patients

Patients in both treatment conditions were treated by the same dentist, a 40-year-old Norwegian woman, who had completed her dental studies in Oslo 12 years prior to the study. Although she had no relevant specialisation, she had completed a theoretical exam on CBT treatment for dental anxiety (equivalent to a 40-h course) and participated in additional video-assisted training in the practical use of CBT, provided by the co-authors. The RCT study was conducted in a dental practice setting that included two other general dental practitioners, two specialists (surgery and endodontics), and one dental hygienist. The clinic had a shared waiting room.

#### Video-evaluation

All sessions were videotaped. A random selection of tapes was evaluated by the co-authors (TW and BT) to assess adherence to manuals in both treatment conditions.

### Background measures

Age, sex, and years since last completed dental treatment were registered at enrolment along with symptoms of anxiety, depression and Post-Traumatic Stress Disorder (PTSD). Self-report scales were employed. All the scales are extensively used worldwide and have been shown to be valid and reliable (see details in the description of each scale).

Participants that reported a traumatic incidence were asked to complete the PTSD checklist for DSM-IV (PCL), version PCL-S. The scale assesses PTSD-symptoms through 17 questions with five possible answers, resulting in a sum score of 17 to 85. Cut-off in a non-clinical population is proposed to be 35 [24]. PCL-S scores were dichotomized accordingly. When dichotomizing scores, patients not reporting a traumatic incidence were assumed to have a score lower than 35.

Symptoms of anxiety and depression were assessed with the Hospital Anxiety and Depression Scale (HADS) developed by Zigmond et al. in 1983 [25]. HADS consists of two subscales; HADS-A and HADS-D, with 7 questions in each meant to measure symptoms of anxiety or depression. Sum score varies from 0–21 in both subscales. For the analysis, scorings on HADS-A and HADS-D were both dichotomized with a cut-off score of 8 or more as established in the literature [26].

### Outcome measures

The change in dental anxiety from pre- to one-year after-treatment was used as a measure of treatment efficiency. Dental anxiety was measured before, after and one year after treatment with the Modified Dental Anxiety Scale (MDAS) and the Index of Dental Anxiety and Fear (IDAF-4C). MDAS mentions 5 potentially frightening stimuli which are scored on a five-point-scale. A sum score (range 5–25) of 15 plus/19 plus indicate high/extremely high dental anxiety [27]. For analytic purposes scores were dichotomized around the established cut off of 19. The IDAF-4C -dental anxiety and fear module, comprises eight items, with two items each measuring the emotional, behavioural, cognitive, and physiological components of anxiety. The items were scored on a 5-point scale, with responses ranging from 1 (‘disagree’) to 5 (‘strongly agree’). As recommended, the median of all items was calculated to obtain an overall score (range: 1–5) [28].

To measure possible negative psychological effects of treatment, the changes in psychometric measures (HADS described above) from pre- to one-year after-treatment were calculated. PTSD-symptoms were compared between start and end of intervention.

The patient-dentist relationship was mapped with the Working Alliance Inventory for clients (WAI) which consists of 12 statements to be evaluated on a 7-item scale. It measures the following dimensions: 1. The relationship 2. The agreement on goals and 3. The agreement on tasks between the therapist and the patient [29]. Average score is reported (Range 1–7). It was decided to report the dimension of WAI that describe the relationship between therapist and patient (Wai-bond) since this dimension was assumed to be most relevant to the dental treatment situation. There is no established cut-off for this scale.

Ten aspects believed to be of importance to treatment success were defined by the authors in collaboration with 2 professors of psychology (Table 2) and the patients were asked to do an evaluation of the items on a 6-point scale as follows: “The dental anxiety treatment involve several different aspects. It would be useful to know more about how some of these aspects have influenced your treatment. Please put a cross on the alternative that fits better for you.”

**Table 2:** Aspects of treatment considered to be of importance to treatment effect

1	To know what was about to happen
2	Experiencing control in the treatment situation
3	Conversations with the dentist
4	The behaviour of the dentist
5	The behaviour of the assistant
6	Absence of pain
7	Increased self-esteem in relation to the dental treatment
8	Gradual progression of the dental treatment
9	Knowledge about anxiety reactions
10	Knowledge about pain

The table describes the aspects of treatment included in the questionnaire “The Patient Evaluation”

### Missing data

Fourteen patients did not complete the treatment. In addition, 5 patient records were lost at the storage facility (see details in Additional file [Additional file 4]). The questionnaires were electronic and completed with access to assistance in the dental office before and after treatment, and at home one year after treatment. Missing input would impede the completion and therefore all questionnaires delivered were complete. However, the WAI was delivered on a different occasion (at the end of the first treatment session) and was not completed by 16 additional patients (a total of 30 patients). PCL-S was completed only by patients reported to have been through a traumatic incidence (*n* = 37) and was omitted at the one-year follow up. Intention-To-Treat analyses were conducted. Imputations of missing data in the follow up measurements were done through the method Last-Observation-Carried-Forward. Last available observations replaced the missing observations, meaning that in those patients that did not complete the study the measurements at baseline were carried forward and treatment effect thus assumed to be zero. Those that did not complete PCL-S at baseline (that had not been through a traumatic event) were not included in the analyses of changes in PCL-S; hence only a completers analysis was available for this measure.

### Statistical analysis

The data was not normally distributed (see additional file for normality tests [Additional file 5]) and statistical significance was determined with the Wilcoxon signed rank test. Differences between groups were calculated with the Mann–Whitney test. In the part of the analyses that investigated how changes in dental anxiety and comorbid symptom scores depended on patient characteristics, the two treatment conditions were treated as one to avoid small group sizes. This was considered appropriate since all available analyses showed comparable findings in both treatment conditions. Multiple testing was done and therefore the *p*-values should be regarded with caution [30]. Scatterplots were also constructed to illustrate how the treatment outcome covaried with comorbid symptoms. Although non-parametric analyses were considered most appropriate, testing with parametric analyses (t-tests) did not change the study outcome. In lack of a better alternative, it was therefore considered applicable to perform calculations of effect sizes with Cohen´s d in order to compare the present study findings with otherwise comparable data in previous research.

All analyses were done using the Stata/SE 16.0 statistical software.

## Results

Participants’ age ranged from 19 to 65 years and 63 (66%) were women. MDAS-scores spanned from 12 to 25. Most patients included scored above cut-off (19) on MDAS (*n* = 82, 85%). The number of years since last dental treatment varied from 0 to 40 years. Half of the patients reported 6 years or more of avoidance of regular dental treatment. Comorbid symptomatology was prevalent: 26% scored above cut-off on symptoms of depression (HADS-D ≥ 8) while 49% were above cut-off on symptoms of anxiety (HADS-A ≥ 8). 54 patients reported to have been through a traumatic life experience and 39% had a PCL-S score of 35 or higher indicating symptoms of PTSD. See Demographics according to treatment group in Table 3.

**Table 3:** Background demographics

	D-CBT (*n* = 48)	FHM (*n* = 48)
Variables		
Age, mean (SD)	38 (13)	39 (12)
Sex, female, n (%)	33 (69%)	30 (63%)
Years since treatment, mean (SD)	11 (10)	9 (9)
MDAS, mean (SD)	21 (3)	21 (3)
IDAF-4C, mean (SD)	4,2 (0,5)	4,1 (0,7)
Traumatic incidence, n (%)	26 (54%)	28 (58%)

The table shows background measures in the intervention group and the control group by means or percentages. The means of measures taken by the Modified Dental Anxiety Scale (MDAS) and Index of Dental Anxiety and Fear, the anxiety and fear module (IDAF-4C), before treatment for each treatment group are found in this table. Years since treatment is the number of years since the patient last received regular (not acute) dental treatment. The number of patients that are female are reported as well as the number of patients that state that they have experienced a traumatic incidence. Standard deviations entre parenthesis for means, percentages entre parenthesis for binominal data. D-CBT: dentist-administered cognitive behavioural therapy. FHM: The Four Habits Model/Midazolam

Only one patient did not accept the randomly selected treatment (FHM) and only four did not complete the full set of appointments due to no-show. Eighty-two patients completed the treatment and the post-treatment questionnaire. Fifty-two responded to the one-year survey and four in five of those that replied to the latter (*n* = 40/52) had continued with dental treatment after participation in the RCT. Four were still on waiting lists for further psychological treatment, while eight had discontinued dental treatment for the following reasons: high dental anxiety (*n* = 4), cost (*n* = 2) and mixed reasons (*n* = 2). See details on study flow in Additional file [Additional file 6].

Treatment effects measured by decrease in dental anxiety were large in both treatment conditions (Table 4). Cohen´s d effect sizes calculated by the amount of change in MDAS-values from pre-treatment and one year after treatment in completers, were identical in the two treatment conditions: d = 1.7 (1.1–2.4). The effect sizes stayed large also when basing the calculation on Intention-To-Treat values: D-CBT: 1.2 (95% CI:0.8–1.7) and Four Habits/midazolam: 1.4 (95% CI:0.9–1.8). The number of patients with extreme dental anxiety decreased from 85% (82/96) before treatment, to 16% (12/77) immediately after treatment and 19% (10/52) one year after treatment. The decline in dental anxiety occurred irrespective of presence of comorbid symptoms of anxiety, depression, or PTSD, and irrespective of dental anxiety level at start of treatment (Table 5).

**Table 4:** Changes in symptoms of depression, anxiety and PTSD during treatment

		**T0**	**T1**	**T2**	**Difference T2-T0**	**Time-effect**	**Group-effect**
		Median	Median	Median	Median	Wilcoxon	Mann-
		(Min,Max)	(Min,Max)	(Min,Max)	(Min,Max)	Signed rank	Whitney test
**MDAS**	D-CBT:	22.0	16.0	15.5	5.0	***p* < 0.001**	
Level of dental	(*n* = 48)	(12,25)	(6,24)	(5,25)	(-1,16)		
anxiety	FHM:	21.0	16.0	15.0	5.5	**p < 0.001**	
	(*n* = 48)	(14,25)	(5,25)	(5,25)	(-3,15)		
**HADS-D**	D-CBT:	3.0	2.5	3.0	0.0	*p* = 0.669	*p* = 0.172
Symptoms of	(*n* = 48)	(0,16)	(0,16)	(0,16)	(-10,7)		
Depression	FHM:	4.0	2.0	2.0	0.5	***p* = 0.015**	
	(*n* = 48)	(0,14)	(0,12)	(0,12)	(-3,7)		
**HADS-A**	D-CBT:	9.0	8.0	7.0	1.0	***p* = 0.005**	*p* = 0.968
Symptoms of	(*n* = 48)	(0,18)	(0,17)	(0,17)	(-7,9)		
Anxiety	FHM:	9.0	7.0	7.0	1.0	***p* = 0.023**	
	(*n* = 48)	(1,20)	(1,18)	(0,17)	(-11,11)		
***PCL-S***	*D-CBT:*	*45.5*	*42.5*	*-*	*4.0*	*p* = 0.200	p = 0.646
*Symptoms of*	*(n* = *26/22/17)*	*(18,67)*	*(19,69)*	*-*	*(-9,23)*		
*PTSD*	*FHM:*	*41.5*	*38.5*	*-*	*0.0*	*p* = 0.762	
	*(n* = *28/24/19)*	*(18,74)*	*(17,57)*	*-*	*(-17,37)*		

Intention to treat analysis of symptoms of anxiety and depression before (T0) and after treatment (T1) as well as one year after treatment (T2). Completers analyses of symptoms of PTSD at T0 and T1. The median values of PCL-S are estimated based on a different number of patients at T0 and T1. (There was no registration of PCL-S values at T2). The number of patients is indicated chronologically entre parenthesis. The difference is calculated between T2 and T0 when measures were available for T2. D-CBT: dentist-administered cognitive behavioural therapy. *FHM* The four habits model/midazolam, *MDAS* Modified dental anxiety scale, *HADS-A* Hospital anxiety and depression scale, anxiety subscale, *HADS-D* Hospital anxiety and depression scale, depression subscale, *PCL-S* The PTSD-checklist for DSM-IV, specific version, *PTSD* Post-traumatic stress disorder

**Table 5:** Reduction in dental anxiety in relation to severe dental anxiety and comorbid symptoms

D-CBT		**T0**	**T1**	**T2**	**Difference T2-T0**	***p***
		Median (Min, Max)	Median (Min,Max)	Median (Min,Max)	Median (Min,Max)	Wilcoxon signed rank
Variables						
**All**	*n* = 48	22.0	16.0	15.5	5.0	***p* < 0.001**
		*(12,25)*	*(6,24)*	*(5,25)*	*(-1,16)*	
**Severe dental**	*MDAS* ≥ *19, n* = *42*	22.0	17.0	16.5	5.0	
**anxiety**		*(19,25)*	*(7,24)*	*(5,25)*	*(-1,16)*	***p* < 0.001**
*n* = *48*	*MDAS* < *19, n* = *6*	15.0	12.0	7.0	6.0	
		*(12,18)*	*(6,16)*	*(5,16)*	*(2,10)*	***p* = 0.030**
**PTSD-**	*PCL-S* ≥ *35, n* = *20*	22.0	18.0	17.5	5.0	
**symptoms**		*(15,24)*	*(11,24)*	*(5,23)*	*(0,15)*	***p* = 0.001**
*n* = *48*	*PCL-S* < *35, n* = *28*	*21.0*	*14.5*	*14.0*	*5.0*	
		*(12,25)*	*(6,23)*	*(5,25)*	*(0,15)*	***p* < 0.001**
**Anxiety-**	*HADS-A* ≥ *8, n* = *21*	22.0	18.0	16.0	6.0	
**symptoms**		*(13,25)*	*(6,24)*	*(5,23)*	*(0,15)*	***p* = 0.001**
*n* = *48*	*HADS-A* < *8, n* = *27*	*22.0*	*15.0*	*15.0*	*5.0*	
		*(12,25)*	*(6,23)*	*(5,25)*	*(-1,16)*	***p* < 0.001**
**Depression-**	*HADS-D* ≥ *8, n* = *12*	22.0	18.0	18.5	4.5	
**symptoms**		*(19,24)*	*(9,22)*	*(8,22)*	*(0,13)*	***p* = 0.010**
*n* = *48*	*HADS-D* < *8, n* = *36*	*22.0*	*15.5*	*14.5*	*5.0*	
		*(12,25)*	*(6,24)*	*(5,25)*	*(-1,16)*	***p* < 0.001**
Four Habits/ Midazolam		**T0**	**T1**	**T2**	**Difference T2-T0**	***P***
		Median	Median	Median	Median	Wilcoxon signed
		(Min,Max)	(Min,Max)	(Min,Max)	(Min,Max)	rank
Variables						
**All**		*21.0*	*16.0*	*15.5*	*5.5*	***p* < 0.001**
		*(14,25)*	*(5,25)*	*(5,25)*	*(-3,15)*	
**Initial**	MDAS ≥ 19, *n* = 40	*21.5*	*16.0*	*15.5*	*5.5*	***p* < 0.001**
**anxiety**		*(19,25)*	*(6,25)*	*(6,25)*	*(-3,15)*	
*n* = 48	MDAS < 19, *n* = 8	*17.0*	*11.0*	*11.0*	*6.0*	***p* = 0.030**
		*(14,18)*	*(5,18)*	*(5,18)*	*(-1,9)*	
**PTSD-**	PCL-S ≥ 35, n = 18	*21.5*	*17.0*	*17.5*	*4.0*	***p* = 0.002**
**symptoms**		*(14,25)*	*(10,24)*	*(9,25)*	*(-2,12)*	
*n* = 48	PCL-s < 35, *n* = 30	*20.5*	*14.5*	*14.0*	*8.0*	***p* < 0.001**
		*(14,25)*	*(5,25)*	*(5,25)*	*(-3,15)*	
**Anxiety-**	HADS-A ≥ 8, *n* = 26	*21.0*	*16.0*	*15.0*	*7.5*	***p* < 0.001**
**symptoms**		*(14,25)*	*(7,24)*	*(5,23)*	*(-1,12)*	
*n* = 48	HADS-A < 8, *n* = 22	*20.0*	*15.5*	*15.5*	*4.5*	***p* < 0.001**
		*(15,25)*	*(5,25)*	*(6,25)*	*(-3,15)*	
**Depression-**	HADS-D ≥ 8, *n* = 13	*22.0*	*17.0*	*15.0*	*5.0*	***p* = 0.003**
**symptoms**		*(14,25)*	*(11,24)*	*(12,22)*	*(-1,11)*	
*n* = 48	HADS-D < 8, *n* = 35	*21.0*	*16.0*	*14.0*	*8.0*	***p* < 0.001**
		*(14,25)*	*(5,25)*	*(5,25)*	*(-3,15)*	

An Intention-To-Treat analysis on outcome of treatment in relation to severity of dental anxiety and comorbid symptoms before treatment. The median values were estimated at T0 (pre-treatment), T1 (post-treatment) and T2 (one-year post-treatment). *D-CBT* Dentist-administered cognitive behavioural therapy, *FHM* The four habits model/midazolam, *HADS-A* hospital anxiety and depression scale, anxiety subscale, *HADS-D* Hospital anxiety and depression scale, depression subscale, *PCL-S* The PTSD-checklist for DSM-IV, specific version, *PTSD* Post-traumatic stress disorder

Symptoms of anxiety, depression and PTSD decreased (Table 6). The decline was larger in patients with increased level of psychological symptoms pre-treatment than in those without. The difference was significant for depression symptoms (*p* < 0.001) as well as for symptoms of PTSD (*p* = 0.039). The decline was however not large enough to reach significance for anxiety symptoms (*p* = 0.095).

**Table 6:** Descriptive Intention-To-Treat (ITT) analyses of changes in anxiety and depressive symptoms and completers analysis in post-traumatic stress disorder symptoms in all patients and in those with elevated symptoms before treatment

	**T0**	**T1**	**T2**	**Difference T2-T0**
	Median	Median	Median	Median
	(Min,Max)	(Min,Max)	(Min,Max)	(Min,Max)
**All**				
HADS-A (anxiety) (*n* = 96)	**9.0**	**7.0**	**7.0**	**1.0**
	(0,20)	(0,18)	(0,17)	(-11,11)
HADS-D (depression) (*n* = 96)	**3.5**	**2.0**	**2.5**	**0.0**
	(0,16)	(0,16)	(0,16)	(-10,7)
PCL-S (PTSD) (*n* = 96)	**44.0**	**40.5**	**-**	**1.0**
	(18,74)	(17,69)	-	(-17,37)
**Anxiety-symptoms**	**10.0**	**9.0**	**9.0**	**2.0**
(HADS-A T0 ≥ 8, *n* = 47)	(8,17)	(0,17)	(2,17)	(-7,9)
**Depression-symptoms**	**10.0**	**9.0**	**8.0**	**2.0**
(HADS-D T0 ≥ 8, *n* = 25)	(8,16)	(1,16)	(1,16)	(-2,7)
**PTSD-symptoms**	**48.0**	**44.0**	**-**	**5.0**
(PCL-S T0 ≥ 35, *n* = 37/25/25)	(36,74)	(29,69)	-	(-16,37)

*T0* Pre-treatment, *T1* Post-treatment, *T2* One-year post-treatment, *D-CBT* Dentist-administered cognitive behavioural therapy, *FHM* The four habits model/midazolam, *HADS-A* Hospital anxiety and depression scale, anxiety subscale, *HADS-D* Hospital anxiety and depression scale, depression subscale, *PCL-S* The PTSD-checklist for DSM-IV, specific version, *PTSD* Post-traumatic stress disorder

In the patient evaluation, all the treatment aspects included were considered by the patients to be of high importance. Aspects relevant to the dentist-patient relationship were estimated to be most important (the behaviour of the dentist and conversations with the dentist) (see Table 7).

**Table 7:** The patient evaluation. Median scores when important aspects of treatment were valued on a scale from 1–6 by the patients

**Important aspects of treatment**		**Median score**	Range
The behaviour of the dentist	1	**6.0**	3–6
Conversations with the dentist	2	**6.0**	3–6
Absence of pain	3	**6.0**	3–6
To know what was about to happen	4	**6.0**	1–6
The behaviour of the assistant	5	**6.0**	1–6
Experiencing control in the treatment situation	6	**6.0**	1–6
Gradual progression of treatment	7	**6.0**	2–6
Knowledge about anxiety reactions	8	**5.0**	1–6
Increased self-esteem in relation to treatment	9	**6.0**	1–6
Knowledge about pain	10	**5.0**	1–6

The relationship scores measured by WAI were very high (median: 6.5, min/max: 3.8,7.0) and did not differ between treatment conditions or according to comorbid symptomatology. This indicates that a very good relationship was established between the dentist and most of the patients. In one third of the patients (*n* = 26) the relationship with the dentist was rated with maximum score (Wai-bond = 7.0), and only two patients reported a score in the lower half of the scale (between 1 and 4). The emergency plan was never put into action during the close to 400 treatment sessions completed. One of the participants experienced increased symptoms of generalized anxiety (GAD) and needed a referral to a psychologist. This increase in GAD was assumed to be provoked by the dental anxiety treatment.

The conclusion from the video evaluation was that, in the selected sessions, the interventions had been accomplished according to the respective treatment manuals.

## Discussion

In the present study comorbid symptoms of anxiety, depression or PTSD did not inhibit the treatment efficiency of two dentist-administered dental anxiety treatment methods. Nor did the treatment delivered coincide with an increase in symptoms of anxiety, depression, or PTSD in neither method. On the contrary, comorbid symptoms decreased during the dental anxiety treatment. Both dentist-administered CBT-treatment and Four Habits Model/midazolam-treatment proved to reduce dental anxiety effectively. The patients evaluated aspects relevant to the dentist-patient relationship to be the most important factors for treatment effect. The available emergency plan in case of acute psychological distress never had to be put into action.

The use of validated instruments renders it possible to compare the present findings with findings in other relevant studies, while the standardized treatment protocols enhance reproducibility. Self-report measures are used in this study as well as in many comparable studies [4]. However, adding performance tests or physiological measures could have added further strength to the study findings. The study does not address all psychiatric comorbidities; hence the findings do not clarify how the treatment conditions might affect patients with symptoms of other psychiatric illnesses. For instance, a report indicated that substance use disorder has potential to influence outcome of dental anxiety treatment negatively [31]. The inclusion of screening for other symptoms of mental illnesses could therefore potentially have rendered different findings. However, as seen in the review by Halonen et al. on the association between dental anxiety and psychiatric disorders and symptoms, symptoms of anxiety, depression and PTSD are very commonly found in the target group [32] and are therefore arguably of relevance. The subgroup analyses necessary to evaluate treatment effects for patients with differing characteristics were planned for in the study design and are strengthened by the uniform findings. However, the sample sizes were small, and this should be kept in mind when evaluating the findings.

Only one dentist treated all the patients. With this study design dentist variability could not influence the findings, which strengthens the inferences that can be drawn from the study. However, at the same time the study design limits the generalizability of the findings to other dentists. Yet, as both treatment methods were delivered with strict adherence to detailed treatment manuals, replication of treatments by other dentists is facilitated.

The presence of drop-out and lost records could bias all repeated scores. The LOCF-design used to replace missing data is conservative and might underestimate study findings. It is a strength to the study that variability of the treatment effects over time was assessed.

The symptoms of PTSD were measured only among patients that reported to have experienced a traumatic event and were removed from the questionnaire one year after treatment. This weakened the findings based on this variable.

The present study confirms the presence of high comorbid symptomatology in patients with strong dental anxiety reported by Halonen et al. [32]. In a British study among patients that attended a UK specialist clinic for dental phobia, mean HADS scores were equivalent to the present findings [33]. Also, the number of dental anxiety patients reporting traumatic life experiences is comparable to what was found in a Dutch study on the effect of dental anxiety treatments in patients with or without a trauma-related sequelae [34]. This indicates that the current sample is representative for patients with severe dental anxiety regarding presence of comorbid psychological symptoms. The lack of a diagnosis may make it difficult to compare the findings in the present study to otherwise comparable studies on dental phobia. In two Norwegian studies by Kvale et al. and Haukebø et al. about half of the patients (primarily self-referred) turned out to qualify for the diagnosis [35, 36]. Thusly, it may be assumed that there was a high presence of patients fulfilling the criteria for dental phobia among the study participants in the present study, even if this could not be assessed with the present study design.

The hypothesis “High score on comorbid symptoms (anxiety, depression, or PTSD) will not negatively affect the dental anxiety treatment effect measured by reduction in MDAS-score” was supported. No association was found between psychological symptoms and the effect of the dental anxiety treatment. This is in line with earlier studies on psychologist-administered or -assisted dental anxiety treatments [34]. Independently of comorbid symptomatology, the treatment effects were large. In a study that reported psychologist assisted dental anxiety treatment in a comparable patient sample, the effect size, estimated from the change in DAS score from pre-treatment to follow-up, was d = 1.6 (no CI reported) [35]. This is comparable to what was found in the present study: d = 1.7 (CI: 1.1–2.4) and indicate that the effect of the dental anxiety treatment administered by a non-specialist dentist in primary care was not inferior to comparable treatments in a specialist clinic involving a psychologist.

The hypothesis “No increase in symptoms of general anxiety, depression, or PTSD will be observed following systematic dental anxiety treatment administered by a dentist in a general dental practice” was supported. This finding contrasts with a recent systematic review in which Bürklein et al. In this paper it is recommended that when high levels of dental anxiety are associated with a long avoidance (> 2 years) a psychologist or psychiatrist should be consulted. This is justified by the authors due to the high frequency of other psychological disorders seen in patients with elevated dental anxiety. However, Bürklein et al. had no findings themselves, or reference to findings by other researchers, that support this recommendation [6].

In the present study the dental anxiety treatment was followed by a decrease in the psychological symptom load. This leans support to previous findings on the effect of dental anxiety treatment on comorbid psychological symptoms. In a Swedish study comparing systematic desensitization by psychologist with sedation treatment in patients with severe dental anxiety (mean avoidance six years), positive generalization effects were reported [14]. A Danish study saw reduced general fearfulness as well as elevated mood in dental anxiety patients that had received desensitization training [37]. In 2000, Vassend et al. showed that dental anxiety treatment by a dentist lead to a significant decline in emotional distress symptoms [10]. The positive findings on comorbid symptoms after dental anxiety treatments appear to be comparable independently of the profession of the person that administered the treatments.

These findings support that dentist-administered treatments could safely serve as a cost-effective first line treatment of dental anxiety of differing severity. This could greatly increase access to treatment for patients in need. In the present study a second line treatment option involving a psychologist was available and referrals were made for about one third of the patients. Educating dentists to do first line treatments for dental anxiety should not be expected to eradicate the need for dental anxiety/dental phobia treatments by psychologist/psychiatrist. It may however relieve the pressure on such specialised services since a lower level of care appears to be satisfactory for the majority of the patients.

The hypothesis “The patient-rated dentist-patient relationship will be of importance to the outcome of treatment by D-CBT and Four Habits/midazolam” was partially supported. Ceiling effects have been reported in relation to the Working Alliance Inventory as well as in other tools for measuring relationship/alliance [38]. This could also be suspected in the present study since the variability of the scores was low. With this distribution of data the comparison of patients with higher and lower scores was not meaningful. However, since the scores were generally high it may still be reasonable to assume that the good dentist-patient relations found in the present study contributed to the favourable outcomes seen. Another finding that supports this assumption was that aspects relevant to the dentist-patient relationship were estimated by the patients to be the most important element of the treatment. This corresponds well with a study by Yuan that showed that dental anxiety was correlated to the dentist communication [20]. In a Norwegian qualitative study on dentists administering CBT-treatments for dental anxiety, it was concluded that a shift of focus is necessary on part of the dentists that do dental anxiety treatments. According to Bryne et al., dentists that aim to treat dental anxiety must look beyond the technicalities of restoring oral pathologies and become able to create a safe space for the patient [39]. Kranstad et al. found that patients with a history of sexually abuse want a stable long-term relationship with a dentist [40]. Use of systematized evidence-based methods in a general dental practice by dentists adequately trained in relational skills may represent a viable treatment option for this patient group. The present findings emphasize the need for further research on aspects related to the dentist-patient relationship and how it influences the outcome of treatment in patients with dental anxiety.

## Conclusions

In conclusion, efficient dental anxiety treatment could be administered by a trained dentist by at least two methods available in a general dental practice. The treatments investigated were not associated with adverse effects and were adequate for patients with severe dental anxiety and for patients with elevated symptoms of anxiety, depression or PTSD.

More detailed research on relations between changes would be interesting, with larger groups of patients with different severities of initial conditions.

Dentists that deliver such treatments need training, including guidance in relational skills. Systematic dental anxiety treatment by a dentist can be effective and important for good oral health in a lifetime perspective. Hence, establishing a best practice for treatment of patients with dental anxiety in general dental practice should be a shared ambition for clinicians, researchers, and educators.

## Supplementary Information

**Additional file 1.** The D-CBT manual. Detailed description of the D-CBT treatment condition.

**Additional file 2.** The Four Habits/midazolam treatment outline.

**Additional file 3.** A comparison of the two treatment conditions, D-CBT and FHM.

**Additional file 4.** The study flow diagram shows the process of inclusion and exclusion of participants as well as drop-out and loss of data during the study course.

**Additional file 5.** Loss of data. The text explains the loss of data due to technical difficulties during the study.

**Additional file 6.** Normality tests. The tables show tests of normality for the data collected in the study.

## Data Availability

The data that support the findings of this study are available from the Dental Faculty at the University of Oslo, but restrictions apply to the availability of these data, which were used under license for the current study, and so are not publicly available. Data are however available from Mariann Saanum Hauge or prof. Tiril Willumsen upon reasonable request and with permission of the Dental Faculty at the University of Oslo.

## Abbreviations

BS: Bent Storå
CBT: Cognitive behavioural therapy
D-CBT: Dentist-administered cognitive behavioural therapy
DSM-IV: The diagnostics and statistical manual for mental disorders, 4^th^ version
FHM: The four habits model/midazolam treatment condition
GAD: Generalized anxiety disorder
HADS: Hospital anxiety and depression scale
HADS-A: Hospital anxiety and depression scale, anxiety subscale
HADS-D: Hospital anxiety and depression scale, depression subscale
ITT: Intention-to-treat
MDAS: The modified dental anxiety scale
PCL-IV: The PTSD checklist for DSM-IV
PCL-S: The PTSD checklist for DSM-IV; specific version
PTSD: Post-traumatic stress disorder
RCT: Randomized controlled trial
TSD: Tjeneste for sensitive data
TW: Tiril Willumsen
WAI: Working alliance inventory
WAI-bond: The WAI-subscale on the bond between operator and patient
