# Supplementary material for: Changes in symptoms of anxiety, depression, and PTSD in an RCT-study of dentist-administered treatment of dental anxiety
Source: BMC Oral Health. 2023 Jun 22;23:415. doi: 10.1186/s12903-023-03061-4 (PMC10288821; doi:10.1186/s12903-023-03061-4)
Supplement: Supplementary file 1 — Additional file 1. The D-CBT manual. Detailed description of the D-CBT treatment condition. [file 12903_2023_3061_MOESM1_ESM.docx]

D-CBT

Practical manual for dentist administered cognitive behavioural therapy for treatment of adult patients

**The treatment is done in 3-5 consultations with a total time consumption of 300 minutes. There should be a maximum interval of 2 weeks between each appointment. All 5 appointments should be scheduled before starting treatment.**

CONTENTS

1. appointment

- - - - - Exploring the patient’s problem
        - The patient perspective: patient wishes and goals
        - Psychoeducation and exploration of individual anxiety

-catastrophic thoughts

-bodily signs of anxiety

-anxiety hierarchy

- - - - - Alliance
        - Method

-anxiety curve

-stop signal

- - - - - Coping techniques/breathing excercise
        - Sum up the appointment, homework, plan for next appointment

2.-4. appointment

- - - - - Daily catastrophic thoughts and bodily signs of anxiety
        - Exposure training
        - Sum up the appointment, homework, plan for next appointment

5. appointment

- - - - - Daily catastrophic thoughts and bodily signs of anxiety
        - Exposure training
        - Treatment summary
        - Plan for further treatment -anxiety and dental needs

Patient number: _____________ Start date: ___________________­­_____

**MODIFIED DENTAL ANXIETY SCALE**

**CAN YOU TELL US HOW ANXIOUS YOU GET, IF AT ALL,**

**WITH YOUR DENTAL VISIT?**

**PLEASE INDICATE BY INSERTING ‘X’ IN THE APPROPRIATE BOX**

| 1. **If you went to your Dentist for TREATMENT TOMORROW, how would you feel?** | | | | | |
| --- | --- | --- | --- | --- | --- |
|  | *Not*  *Anxious* ⬜ | *Slightly*  *Anxious* ⬜ | *Fairly*  *Anxious* ⬜ | *Very*  *Anxious* ⬜ | *Extremely*  *Anxious* ⬜ |
|  | | | | | |
| 1. **If you were sitting in the WAITING ROOM (waiting for treatment), how would you feel?** | | | | | |
|  | *Not*  *Anxious* ⬜ | *Slightly*  *Anxious* ⬜ | *Fairly*  *Anxious* ⬜ | *Very*  *Anxious* ⬜ | *Extremely*  *Anxious* ⬜ |
|  | | | | | |
| 1. **If you were about to have a TOOTH DRILLED, how would you feel?** | | | | | |
|  | *Not*  *Anxious* ⬜ | *Slightly*  *Anxious* ⬜ | *Fairly*  *Anxious* ⬜ | *Very*  *Anxious* ⬜ | *Extremely*  *Anxious* ⬜ |
|  | | | | | |
| 1. **If you were about to have your TEETH SCALED AND POLISHED, how would you feel?** | | | | | |
|  | *Not*  *Anxious* ⬜ | *Slightly*  *Anxious* ⬜ | *Fairly*  *Anxious* ⬜ | *Very*  *Anxious* ⬜ | *Extremely*  *Anxious* ⬜ |
|  | | | | | |
| 1. **If you were about to have a LOCAL ANAESTHETIC INJECTION in your gum, above an upper back tooth, how would you feel?** | | | | | |
|  | *Not*  *Anxious* ⬜ | *Slightly*  *Anxious* ⬜ | *Fairly*  *Anxious* ⬜ | *Very*  *Anxious* ⬜ | *Extremely*  *Anxious* ⬜ |

© 2020 The University of St Andrews

HUMPHRIS GM, MORRISON T and LINDSAY SJE (1995) 'The Modified Dental Anxiety Scale: Validation and United Kingdom Norms' Community Dental Health, 12, 143-150.

**Index of Dental Anxiety and Fear, the Phobia Module**

Armfield, J. M. (2010). "Development and psychometric evaluation of the Index of Dental Anxiety and Fear (IDAF-4C+)." Psychol Assess **22**(2): 279-287.

These questions are a good starting point for further exploration of individual anxiety.

Mapping the patient’s problem -background, duration, severity

1. **What do you think is the reason that you fear to go to the dentist (special events)?**

|  |
| --- |

1. **When did you start to fear dental treatment? What happened?**

|  |
| --- |

1. **When was the last time you saw a dentist? When was the last time you had finished all necessary dental treatment?**

|  |
| --- |

1. **Have you ever experienced other painful life events that might have affected your dental anxiety? This could be for example bullying, violence, sexual assault, event in hospital?**

|  |
| --- |

1. **Do you have traumatic memories that come back to you when you are in the dental chair?**

|  |
| --- |

1. **Have you ever received treatment for dental anxiety of any kind -with medication or psychological? In the case that you have, what did you receive? Did it work well for you?**

|  |
| --- |

1. **Are there other situations that you fear? Or have feared? In the case there are, -what is it? Have you received any treatment for this?**

|  |
| --- |

Discovering the patient´s wishes and goals

1. **What is your goal with this treatment?**

|  |
| --- |

Psychoeducation

Bodily reactions and catastrophic thoughts

1. **How does your fear of dental treatment manifest itself? What does it feel like in your body?**

|  |
| --- |

*Examples:*

*Bodily increased heart rate, tight muscles, sweat, different breathing*

*Cognitive difficult to think clearly*

Common bodily signs of anxiety and fear are explained through their value in survival. Put this into context with the patient´s individual reactions. Explain fight/flight/freeze. Explain how anxiety reactions affect cognitive function.

1. **Do you have thoughts about bad things that might happen when you receive dental treatment? What is the worst that could happen? What are you the most afraid of?**

|  |
| --- |

Explore individual catastrophic thoughts, follow the thought to the utmost consequence with the patient, and do a reality check: “Is this likely?” Explain how catastrophic thoughts increase the anxiety.

*Examples: die, faint, go crazy, have terrible pain, run out, hit the dentist, that the needle will break, fear of being anesthetized in the brain, broken needle tip wanders to the brain.*

Anxiety hierarchy

1. **Can you grade different parts of the treatment situation according to each other depending of what you fear more or less?**

|  |  |  |  |  |  |  |
| --- | --- | --- | --- | --- | --- | --- |
|  |  |  |  |  |  |  |
|  |  |  |  |  |  |  |
|  |  |  |  |  |  |  |
|  |  |  |  |  |  |  |
|  |  |  |  |  |  |  |
|  |  |  |  |  |  |  |
|  |  |  |  |  |  |  |
|  |  |  |  |  |  |  |
|  |  |  |  |  |  |  |
|  |  |  |  |  |  |  |

Write with a pencil to allow for changes along the way. Start with what scares the most, continue with what scares less, and in the end place what is in the middle. It is important that the patient has an active part in this work.

*Examples: Drilling with anaesthetics, putting anaesthetic, probing teeth, take an x-ray, having a suction in the mouth, cleaning calculus, do an examination by mirror*

Method and coping techniques

Underline main principles of further treatment:

| **Control (explain the procedures, ask for permission, stop by sign)**  **Gradual exposure**  **Minimizing pain** |
| --- |

1. **How do you think it is better for you to signal us to stop? Is it ok for you to lift you left hand? Is something else easier?**

|  |
| --- |

Insctructions for relaxation by breathing**:**

1. **Breath in deeply. Count slowly to four while exhaling. Try first by counting out loud, thereafter by counting inside. Does this make you feel calmer?**

Anxiety curve

The concepts below are explained through handmade drawings

- When fear rises
- Escape
- Staying within the situation
- The window of tolerance

| 10 |  |
| --- | --- |
| 9 |  |
| 8 |  |
| 7 |  |
| 6 |  |
| 5 |  |
| 4 |  |
| 3 |  |
| 2 |  |
| 1 |  |
| 0 |  |

1. **What level of anxiety do you think is acceptable to you during treatment?**

**__________________**

Summarizing today´s appointment (appointment nr. 1)

1. **How was this appointment?**
2. **What was the part of treatment responsible for making it a good/bad experience?**

|  |
| --- |

Alliance

1. **Do I treat you with respect? Do you feel that I take you seriously?**
2. **Do you feel that we are a team (working against the problem)?**

|  |
| --- |

Homework

|  |
| --- |

**Examples: breathing, se movie clip or photo of syringe or drill, talk to someone about the anxiety, find out more about individual catastrophic thoughts**

Plan for next appointment (appointment nr. 2)

|  |
| --- |

**The page is sent with the patient either by taking a photo with cell phone or by photocopying the page.**

**Date: ____________**

Introduction to appointment 2 Summarizing the patient´s anxiety in between session and preparing today´s session:

1. **Have you had any bodily signs of anxiety preceding this appointment? How did that feel?**
2. **Have you had any thoughts about today and about what is going to happen? Can you tell me more about this?**
3. **Are you ready to do what we planned in the last appointment?**

|  |
| --- |

Exposure: shown and explained the following (put a cross at what has been demonstrated).

|  | Dental chair |  |  | LED curing light |
| --- | --- | --- | --- | --- |
|  | **Operation lamp** |  |  | **Filling materials and accessories** |
|  | **Suction** |  |  | **Instruments for examination** |
|  | **Instrument holder** |  |  | **Instruments for doing fillings** |
|  | **Three-way springe** |  |  | **Matrix-system** |
|  | **Handpiece** |  |  | **Syringe** |
|  | **Turbine** |  |  | **Impression accessories** |
|  | **Airscaler** |  |  | **Instruments for extraction** |
|  | **Burs** |  |  | **OPG** |

| Exposure, activities | Anxiety level start | Anxiety level  end |
| --- | --- | --- |
|  |  |  |
|  |  |  |
|  |  |  |
|  |  |  |

Repeated the following aspects of psychoeducation (put a cross at what has been repeated):

|  | Anxiety hierarchy |  |  | Catastrophic thoughts |
| --- | --- | --- | --- | --- |
|  | **Anxiety curve** |  |  | **Bodily signs of anxiety** |
|  | **Window of tolerance** |  |  | **Fight, flight, freeze** |
|  | **Gradual exposure** |  |  |  |

Was a relaxation technique applied? No Yes, breathing Yes, other technique:

Summarizing today´s appointment (appointment nr. 2)

1. **How was this appointment?**
2. **What was the part of treatment responsible for making it a good/bad experience?**

|  |
| --- |

Alliance

1. **Do I treat you with respect? Do you feel that I take you seriously?**
2. **Do you feel that we are a team (against the problem)?**

|  |
| --- |

Homework

|  |
| --- |

**Examples: breathing, se movie clip or photo of syringe or drill, talk to someone about the anxiety, find out more about individual catastrophic thoughts**

Plan for next appointment (appointment nr. 3)

|  |
| --- |

**The page is sent with the patient either by taking a photo with cell phone or by photocopying the page.**

**Date: ____________**

Introduction to appointment 3 Summarizing the patient´s anxiety in between session and preparing today´s session:

1. **Have you had any bodily signs of anxiety before this appointment? How did that feel?**
2. **Have you had any thoughts about today and about what is going to happen? Can you tell me more about this?**
3. **Are you ready to do what we planned in the last appointment?**

|  |
| --- |

Exposure: shown and explained the following (put a cross at what has been demonstrated).

|  | Dental chair |  |  | LED curing light |
| --- | --- | --- | --- | --- |
|  | **Operation lamp** |  |  | **Filling materials and accessories** |
|  | **Suction** |  |  | **Instruments for examination** |
|  | **Instrument holder** |  |  | **Instruments for doing fillings** |
|  | **Three-way springe** |  |  | **Matrix-system** |
|  | **Handpiece** |  |  | **Syringe** |
|  | **Turbine** |  |  | **Impression accessories** |
|  | **Airscaler** |  |  | **Instruments for extraction** |
|  | **Burs** |  |  | **OPG** |

| Exposure, activities | Anxiety level start | Anxiety level  end |
| --- | --- | --- |
|  |  |  |
|  |  |  |
|  |  |  |
|  |  |  |

Repeated the following aspects of psychoeducation (put a cross at what has been repeated):

|  | Anxiety hierarchy |  |  | Catastrophic thoughts |
| --- | --- | --- | --- | --- |
|  | **Anxiety curve** |  |  | **Bodily signs of anxiety** |
|  | **Window of tolerance** |  |  | **Fight, flight, freeze** |
|  | **Gradual exposure** |  |  |  |

Was a relaxation technique applied? No Yes, breathing Yes, other technique:

Summarizing today´s appointment (appointment nr. 3)

1. **How was this appointment?**
2. **What was the part of treatment responsible for making it a good/bad experience?**

|  |
| --- |

Alliance

1. **Do I treat you with respect? Do you feel that I take you seriously?**
2. **Do you feel that we are a team (against the problem)?**

|  |
| --- |

Homework

|  |
| --- |

**Examples: breathing, se movie clip or photo of syringe or drill, talk to someone about the anxiety, find out more about individual catastrophic thoughts**

Plan for next appointment (appointment nr. 4)

|  |
| --- |

**The page is sent with the patient either by taking a photo with cell phone or by photocopying the page.**

**Date: ____________**

Introduction to appointment 4 Summarizing the patient´s anxiety in between session and preparing today´s session:

1. **Have you had any bodily signs of anxiety before this appointment? How did that feel?**
2. **Have you had any thoughts about today and about what is going to happen? Can you tell me more about this?**
3. **Are you ready to do what we planned in the last appointment?**

|  |
| --- |

Exposure: shown and explained the following (put a cross at what has been demonstrated).

|  | Dental chair |  |  | LED curing light |
| --- | --- | --- | --- | --- |
|  | **Operation lamp** |  |  | **Filling materials and accessories** |
|  | **Suction** |  |  | **Instruments for examination** |
|  | **Instrument holder** |  |  | **Instruments for doing fillings** |
|  | **Three-way springe** |  |  | **Matrix-system** |
|  | **Handpiece** |  |  | **Syringe** |
|  | **Turbine** |  |  | **Impression accessories** |
|  | **Airscaler** |  |  | **Instruments for extraction** |
|  | **Burs** |  |  | **OPG** |

| Exposure, activities | Anxiety level start | Anxiety level  end |
| --- | --- | --- |
|  |  |  |
|  |  |  |
|  |  |  |
|  |  |  |

Repeated the following aspects of psychoeducation (put a cross at what has been repeated):

|  | Anxiety hierarchy |  |  | Catastrophic thoughts |
| --- | --- | --- | --- | --- |
|  | **Anxiety curve** |  |  | **Bodily signs of anxiety** |
|  | **Window of tolerance** |  |  | **Fight, flight, freeze** |
|  | **Gradual exposure** |  |  |  |

Was a relaxation technique applied? No Yes, breathing Yes, other technique:

Summarizing today´s appointment (appointment nr. 4)

1. **How was this appointment?**
2. **What was the part of treatment responsible for making it a good/bad experience?**

|  |
| --- |

Alliance

1. **Do I treat you with respect? Do you feel that I take you seriously?**
2. **Do you feel that we are a team (against the problem)?**

|  |
| --- |

Homework

|  |
| --- |

**Examples; breathing, se movie clip or photo of syringe or drill, talk to someone about the anxiety, find out more about individual catastrophic thoughts**

Plan for next appointment (appointment nr. 5)

|  |
| --- |

**The page is sent with the patient either by taking a photo with cell phone or by photocopying the page.**

**Date: ____________**

Introduction to appointment 5 Summarizing the patient´s anxiety in between session and preparing today´s session:

1. **Have you had any bodily signs of anxiety before this appointment? How did that feel?**
2. **Have you had any thoughts about today and about what is going to happen? Can you tell me more about this?**
3. **Are you ready to do what we planned in the last appointment?**

|  |
| --- |

Exposure: shown and explained the following (put a cross at what has been demonstrated).

|  | Dental chair |  |  | LED curing light |
| --- | --- | --- | --- | --- |
|  | **Operation lamp** |  |  | **Filling materials and accessories** |
|  | **Suction** |  |  | **Instruments for examination** |
|  | **Instrument holder** |  |  | **Instruments for doing fillings** |
|  | **Three-way springe** |  |  | **Matrix-system** |
|  | **Handpiece** |  |  | **Syringe** |
|  | **Turbine** |  |  | **Impression accessories** |
|  | **Airscaler** |  |  | **Instruments for extraction** |
|  | **Burs** |  |  | **OPG** |

| Exposure, activities | Anxiety level start | Anxiety level  end |
| --- | --- | --- |
|  |  |  |
|  |  |  |
|  |  |  |
|  |  |  |

Repeated the following aspects of psychoeducation (put a cross at what has been repeated):

|  | Anxiety hierarchy |  |  | Catastrophic thoughts |
| --- | --- | --- | --- | --- |
|  | **Anxiety curve** |  |  | **Bodily signs of anxiety** |
|  | **Window of tolerance** |  |  | **Fight, flight, freeze** |
|  | **Gradual exposure** |  |  |  |

Was a relaxation technique applied? No Yes, breathing Yes, other technique:

Summarizing todays appointment (appointment nr. 5)

1. **How was this appointment?**
2. **What was the part of treatment responsible for making it a good/bad experience?**

|  |
| --- |

Alliance

1. **Do I treat you with respect? Do you feel that I take you seriously?**
2. **Do you feel that we are a team (against the problem)?**

|  |
| --- |

Summarize all treatment and plan for further treatment:

1. **What is easier to cope with in the dental treatment situation now compared to when treatment started?**

|  |
| --- |

1. **What is important to you for further treatment? What can the dentist and other dental health personnel do to make dental treatment as comfortable as possible for you?**

|  |
| --- |

Examples: stop-signal, method for anaesthesia, breaks, sitting position, counting, music

1. **Plan for further treatment (both anxiety and dental treatment must be addressed)**

|  |
| --- |

**Additional file 1: The D-CBT manual (above) detailly describe the treatment given in the D-CBT treatment condition**
