# Supplementary material for: Changes in symptoms of anxiety, depression, and PTSD in an RCT-study of dentist-administered treatment of dental anxiety
Source: BMC Oral Health. 2023 Jun 22;23:415. doi: 10.1186/s12903-023-03061-4 (PMC10288821; doi:10.1186/s12903-023-03061-4)
Supplement: Supplementary file 2 — Additional file 2. The Four Habits/midazolam treatment outline. [file 12903_2023_3061_MOESM2_ESM.docx]

Midazolam/The Four Habits model

Treatment outline

**The treatment follows the structure outlined in The Four Habits model:**

- - Investment in the beginning
    - Create rapport quickly
    - Elicit the patients concerns
    - Plan the visit with the patient
  - Elicit the Patient´s Perspective
    - Ask for the patient´s ideas
    - Elicit specific requests
    - Explore the impact on the patient´s life
  - Demonstrate Empathy
    - Be open to the patient´s emotions
    - Make an empathic statement
    - Convey empathy nonverbally
  - Invest in the End
    - Deliver diagnostic information
    - Provide education
    - Involve the patient in making decisions

**In addition, common sense principles are used:**

- - Explain-ask-show-do
  - Start- and stop-signals
  - Minimalize pain during treatment
  - Focus on breathing when considered useful
  - Continuous procedural information
  - Hide/diminish scary stimuli (syringe, other..)
  - Explain anxiety symptoms when considered useful

**Time frame**

60min + 90 min + 90 min + 60 min (300 minutes in 4 appointments).

**1^st^ Appointment**

- 1. **Patient history -dental anxiety**
  2. **Detailed medical history with additional information for sedation (ASA-classification)**
  3. **Patient information about midazolam (written) -fasting, companion, side-effects**
  4. **Informed Consent (written)**
  5. **Necessary x-rays (when possible)**
  6. **Clinical examination (when possible)**
  7. **Treatment plan (short and long term)**

**2^nd^ and 3^rd^ Appointment**

- 1. **Recapitulate the plan for the appointment**
  2. **Sedation form (fasting, companion, O2 saturation, breathing frequency)**
  3. **Patient is given 1 crushed tablet of Dormicum 7.5 mg with water, if moderate effect after 30 minutes another 0,5 tablet is offered**
  4. **Dentist/Dental assistant stays with the patient at all times**
  5. **Continuous monitoring of O2 saturation and breathing**
  6. **Dental treatment starts when the patients states that he/she is ready**
  7. **A blanket is offered to keep the patient warm when sedated**
  8. **If necessary, call the patient a day after the appointment to summarize what happened during the appointment**

**4^th^ Appointment**

1. **The treatment is summed up and evaluated with the patient**
2. **Construct a “Coping plan” to use in further dental treatment**
3. **A further treatment plan for dental and/or dental anxiety treatment is made**
4. **A referral is made when necessary -for dental anxiety treatment, or dental treatment**

**Additional file 2: The Four Habits/midazolam treatment outline (above) describes important treatment principles as well as the treatment outline of the treatment given in the Four Habits/midazolam treatment condition**
