# Supplementary material for: Changes in symptoms of anxiety, depression, and PTSD in an RCT-study of dentist-administered treatment of dental anxiety
Source: BMC Oral Health. 2023 Jun 22;23:415. doi: 10.1186/s12903-023-03061-4 (PMC10288821; doi:10.1186/s12903-023-03061-4)
Supplement: Supplementary file 3 — Additional file 3. A comparison of the two treatment conditions, D-CBT and FHM. [file 12903_2023_3061_MOESM3_ESM.docx]

| **Similarities between Four Habits/midazolam and D-CBT** | |
| --- | --- |
| **History of dental anxiety** | The interview on dental anxiety was semi-structured in D-CBT and unstructured in Four Habits Model/midazolam, but the two are still comparable in time spent and content |
| **Relationship** | Emphasis is put on the creation of a safe and trusting relationship |
| **Communication style** | The Four Habits Model -including ample information about the dental procedures and what to expect from treatment |
| **Patient-centred** | The patient is involved in all decisions to assure treatment progression in accordance with patients’ needs and wishes |
| **Minimalizing pain** | Care is taken to minimize pain inflicted by the dental treatment |
| **Coping plan** | A coping plan is created to formalise the needs and wishes of the patient, and the need for adaptions of further dental treatment |

**Additional file 3: The table shows a comparison of the two treatment conditions, D-CBT (dentist-administered cognitive behavioural therapy and Four Habits Model/midazolam (dental treatment while sedated with midazolam).**
