# Supplementary material for: Changes in symptoms of anxiety, depression, and PTSD in an RCT-study of dentist-administered treatment of dental anxiety
Source: BMC Oral Health. 2023 Jun 22;23:415. doi: 10.1186/s12903-023-03061-4 (PMC10288821; doi:10.1186/s12903-023-03061-4)
Supplement: Supplementary file 4 — Additional file 4. The study flow diagram shows the process of inclusion and exclusion of participants as well as drop-out and loss of data during the study course. [file 12903_2023_3061_MOESM4_ESM.docx]

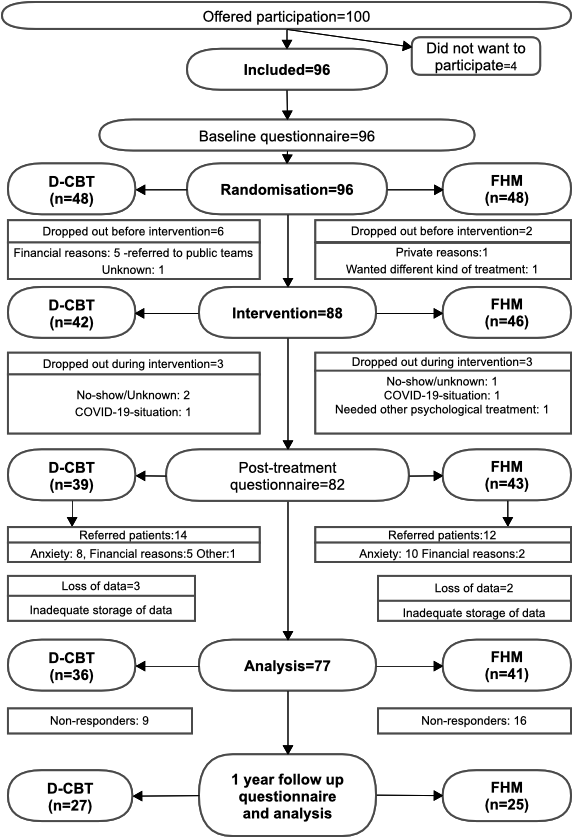


**Additional file 4: The study flow diagram shows the process of inclusion and exclusion of participants as well as drop-out and loss of data during the study course.**
