# Supplementary material for: Changes in symptoms of anxiety, depression, and PTSD in an RCT-study of dentist-administered treatment of dental anxiety
Source: BMC Oral Health. 2023 Jun 22;23:415. doi: 10.1186/s12903-023-03061-4 (PMC10288821; doi:10.1186/s12903-023-03061-4)
Supplement: Supplementary file 5 — Additional file 5. Loss of data. The text explains the loss of data due to technical difficulties during the study. [file 12903_2023_3061_MOESM5_ESM.docx]

**Loss of data**

Data collection and storage was on the TSD (Tjeneste for Sensitive Data) facilities, owned by the University of Oslo, operated, and developed by the TSD service group at the University of Oslo, IT-Department (USIT).

During an update of the services in the spring of 2019 some records were accidentally deleted. When trying to retrieve the data some months later it turned out that also the back-up had been deleted.

This loss of data (post-treatment registrations of five patients) was completely at random.

**Additional file 5 The text explains the loss of data due to technical difficulties during the study.**
