# Supplementary material for: Changes in symptoms of anxiety, depression, and PTSD in an RCT-study of dentist-administered treatment of dental anxiety
Source: BMC Oral Health. 2023 Jun 22;23:415. doi: 10.1186/s12903-023-03061-4 (PMC10288821; doi:10.1186/s12903-023-03061-4)
Supplement: Supplementary file 6 — Additional file 6. Normality tests. The tables show tests of normality for the data collected in the study. [file 12903_2023_3061_MOESM6_ESM.docx]

|  | Shapiro-Wilk test for normal data | | |  |  |
| --- | --- | --- | --- | --- | --- |
|  |  |  |  |  |  |
| **Pre** | Obs | W | V | z | Prob>z |
| **HADS-D (depression)** | 96 | 0.92 | 6.38 | 4.10 | 0.000 |
| **HADS-A (anxiety)** | 96 | 0.99 | 0.91 | -0.21 | 0.585 |
| **PCL-S (PTSD)** | 54 | 0.98 | 1.13 | 0.27 | 0.394 |
| **MDAS (dental anxiety)** | 96 | 0.93 | 0.57 | 3.70 | 0.000 |
| **Wai-bond (relationship)** | 77 | 0.89 | 7.28 | 4.34 | 0.000 |
|  |  |  |  |  |  |
| **Post** |  |  |  |  |  |
| **HADS-D (depression)** | 77 | 0.89 | 7.09 | 4.28 | 0.000 |
| **HADS-A (anxiety)** | 77 | 0.98 | 1.12 | 0.25 | 0.400 |
| **PCL-S (PTSD)** | 46 | 0.99 | 0.57 | -1.18 | 0.882 |
| **MDAS (dental anxiety)** | 77 | 0.99 | 0.92 | -0.19 | 0.574 |
|  |  |  |  |  |  |
|  |  |  |  |  |  |
|  |  |  |  |  |  |
|  | Skewness and kurtosis test for normality |  |  |  |  |
|  |  |  |  | Joint test |  |
| **Pre** | Obs | Pr  (skewness) | Pr (kurtosis) | Adj chi2(2) | Prob>chi2 |
| **HADS-D (depression)** | 96 | 0.003 | 0.582 | 8.03 | 0.018 |
| **HADS-A (anxiety)** | 96 | 0.441 | 0.192 | 2.36 | 0.307 |
| **PCL-S (PTSD)** | 54 | 0.498 | 0.381 | 1.28 | 0.732 |
| **MDAS (dental anxiety)** | 96 | 0.001 | 0.150 | 11.80 | 0.003 |
| **Wai-bond (relationship)** | 77 | 0.001 | 0.852 | 9.64 | 0.008 |
|  |  |  |  |  |  |
| **Post** |  |  |  |  |  |
| **HADS-D (depression)** | 77 | 0.001 | 0.621 | 10.11 | 0.006 |
| **HADS-A (anxiety)** | 77 | 0.238 | 0.075 | 4.60 | 0.100 |
| **PCL-S (PTSD)** | 46 | 0.437 | 0.979 | 0.62 | 0.732 |
| **MDAS (dental anxiety)** | 77 | 0.690 | 0.196 | 1.89 | 0.003 |

**Additional file 6: The tables show tests of normality for the data collected in the study.**
